# Rationale and design of the randomised controlled trial to assess the impact of liraglutide on cardiac function and structure in young adults with type 2 diabetes (the LYDIA study)

**DOI:** 10.1186/s12933-016-0421-6

**Published:** 2016-07-21

**Authors:** Z. Z. Htike, T. Yates, E. M. Brady, D. Webb, L. J. Gray, D. Swarbrick, G. P. McCann, K. Khunti, M. J. Davies

**Affiliations:** NIHR Leicester-Loughborough Diet, Lifestyle and Physical Activity Biomedical Research Unit, Leicester Diabetes Centre, Leicester General Hospital, Leicester, UK and Health Sciences, University of Leicester, Leicester, UK; Department of Cardiovascular Sciences, Glenfield Hospital, University of Leicester, Leicester, UK; NIHR Leicester Cardiovascular Biomedical Research Unit, Glenfield Hospital, Leicester, UK

**Keywords:** Type 2 diabetes, Liraglutide, Cardiac MRI, Cardiac function

## Abstract

**Background:**

The prevalence of type 2 diabetes (T2DM) in younger adults is growing. Compared to the late onset T2DM, it is well recognized that the disease tends to behave more aggressively in the younger age group with evidence of premature micro and macrovasular diseases and shorter life span. This increased mortality is largely attributed to cardiovascular complications. In a recent pilot study, young adults with T2DM were found to have significantly lower peak diastolic strain rate (PEDSR) on cardiac MRI (CMR), a forerunner of diabetic cardiomyopathy. Liraglutide, a glucagon like peptide-1 (GLP-1) analogue, is one of the new classes of glucose lowering therapies licensed to be used in management of T2DM. In randomised controlled trials, liraglutide improves glycaemic control by 1–1.5 % with an added benefit of weight loss of 2–3 kg. In addition, there is emerging evidence elucidating the cardioprotective effects of GLP-1 analogues independent of glycaemic control. In a small study, liraglutide has also been shown to improve cardiac function in patients with coronary ischaemia or congestive heart failure.

**Methods and aims:**

This is a prospective, randomised, open-label, blind end-point (PROBE) active-comparator trial. A total of 90 obese eligible participants with T2DM (18–50 years) will be randomised to either liraglutide 1.8 mg once daily or sitagliptin 100 mg once daily for 26 weeks. The primary aim is to assess whether liraglutide improves diastolic function compared to sitagliptin as measured by PEDSR using CMR.

**Discussion:**

Although newer classes of GLP-1 analogues are made available in recent years, there are very few published studies demonstrating the beneficial effect of GLP-1 analogues on cardiovascular endpoints. In a recently published LEADER study, liraglutide has shown superiority to placebo in a population of type 2 diabetes with high risk of cardiovascular disease. To the best of our knowledge, there are no published studies establishing the effect of liraglutide on cardiac function in younger patients with T2DM on a larger scale. The LYDIA study will comprehensively describe changes in various parameters of cardiac structure and function in patients treated with liraglutide aiming to provide new evidence on effect of liraglutide on diastolic function in young obese people with T2DM.

*Trial Registration* ClinicalTrials.gov identifier: NCT02043054

**Electronic supplementary material:**

The online version of this article (doi:10.1186/s12933-016-0421-6) contains supplementary material, which is available to authorized users.

## Background

### T2DM in young adults and CVD

In the last two decades, the age of onset of type 2 diabetes (T2DM) has decreased with a sharp rise in prevalence of T2DM under 40 years of age [1, 2]. Emerging evidence has shown that increased obesity along with increased sedentary life styles have contributed to the downward shift in age of onset of this condition. In the US, the observed 70 % increase in obesity in young adults (18–29 years) remarkably parallels a 70 % rise in T2DM in adults under 40 years of age [3]. The typical phenotype of obesity, signs of insulin resistance and prevalence of cardiovascular risk factors such as hypertension and dyslipidaemia are very common in this subset of patients. In younger adults, T2DM tends to behave more aggressively with an evidence of premature micro and macrovasular diseases [4] despite the young age and a relatively short duration of diabetes [5].

There is increasing evidence in the literature that developing T2DM in a younger age is a high risk condition for premature atherosclerotic [6] changes and premature cardiovascular disease (CVD) [7]. Although the absolute risk of CVD was reportedly higher in older adults with or without diabetes, younger adults with early-onset T2DM have a higher risk compared to age-matched controls. The risk of developing a myocardial infarction in young adults with T2DM is 14-fold higher than control subjects compared to fourfold higher in those with late onset T2DM [8].

Chronic heart failure (CHF) is a major cause of premature mortality in T2DM. Data from the National Diabetes Audit 2011–12 (NDA), which includes data on almost 2 million patients with diabetes, has shown that CHF was second only to end stage renal disease as a cause of death, and diabetic patients with heart failure were four times more likely to die prematurely than those without [9]. Over one quarter (28 %) of hospitalisations for CHF in the UK (716,106) had diabetes, despite a prevalence of only 5 % in the general population [10]. The NDA concluded that preventing, detecting and treating CHF should become a diabetes management priority.

### Diastolic dysfunction in T2DM

Although the majority of patients with CHF have impaired left ventricular systolic function with reduced ejection fraction (EF), there is increasing awareness of patients with CHF with a normal EF (HF-NEF), particularly in primary care [11]. Patients with HF-NEF have abnormalities of left ventricular relaxation (diastolic dysfunction). There is a high prevalence of diabetes in both forms of CHF. Recent data from our centre has found almost half of patients with HF-NEF (48/101) had diabetes (unpublished data).

It has long been appreciated that patients with diabetes can have heart failure even in the absence of coronary artery disease, hypertension or valvular heart disease, described as diabetic cardiomyopathy [11, 12]. According to Maisch’s proposed classification, asymptomatic diastolic dysfunction with preserved ejection fraction is the earliest stage of diabetic cardiomyopathy [13]. In T2DM, the prevalence of left ventricular diastolic dysfunction varies between 35 and 60 % [14–16]. Adults with diastolic dysfunction are known to be at increased risk of progressing to heart failure if there is co-existing CVD or other cardiovascular risk factors are not well controlled [17].

We and others have shown that adolescents and young adults with diabetes already have evidence of diastolic dysfunction compared to age matched healthy controls [18, 19]. Using cardiac MRI (CMR) our group has shown that peak early diastolic strain rate (PEDSR) is approximately 10 % lower in young adults (18–40) with T2DM compared to obese controls, and 20 % lower compared to lean controls. There was also a strong trend towards reduced systolic strain in T2DM (21 %) compared to both control groups (23 % p = 0.08), suggesting these patients may be on the verge of developing systolic as well as diastolic impairment. Therefore sub-clinical abnormalities in diastolic function are already present in young adults with T2DM, despite a relatively short duration of disease, which may predispose these patients to heart failure.

The mechanism causing these abnormalities is unclear but in our study duration of diabetes and arterial stiffness were both associated with PEDSR [18]. Other proposed causes of diastolic dysfunction in T2DM include vascular, inflammatory, epigenetic, and neuroendocrine mechanisms, as well as myocardial steatosis. We did not see an association of diastolic dysfunction with myocardial perfusion reserve or HbA1c. However, other studies suggest a relationship between glycaemic control and diastolic dysfunction [20, 21]. Therefore, therapeutic strategies that simultaneously target both glycaemic control and diastolic cardiac function will be desirable in younger adults with T2DM.

### GLP therapies and CV function

Based on enhancing the effect of naturally occurring gut hormone Glucagon like peptiede-1 (GLP-1), novel therapeutic agents have been developed and made available in the management of T2DM in the last decade. GLP-1 is secreted from the small intestine in response to oral glucose ingestion and is a potent stimulator of insulin secretion [22]. Physiologically, the action of GLP-1 is short-lived due to rapid inactivation by the dipeptidyl peptidase-4 (DPP-4) enzyme inhibitor.

Incretin based therapies either mimic GLP-1 action because they are structurally similar to GLP-1 (GLP-1 analogues) or they act to reduce the breakdown of GLP-1 by the inhibition of DPP-4 (DPP-4 inhibitors) [23]. In addition to their comparable glucose lowering effects to existing therapies, these new agents offer the advantage of having a low risk of hypoglycaemia and either weight loss (GLP-1 analogue) or weight neutrality (DPP-4 inhibitor).

Emerging experimental evidence suggest that GLP-1 therapy has specific cardioprotective effects independent of weight loss or improvement in glycaemic control. The cardio-protective effects are postulated to be mediated via both the GLP-1 receptor (GLP-1R) dependent and independent pathways involving nitric oxide mediated vasodilatory pathways, reduction of inflammatory mediators such as hs-CRP, monocytes and macrophages etc. [24, 25] .

In particular, GLP-1 treatment has been shown to have direct effects on cardiac function and structure. In small scale animal and human studies, infusion of GLP-1 analogue improves coronary blood flow and left ventricle (LV) function in those with chronic heart failure or after CABG and angioplasty following myocardial ischaemia [26–28]. In rat and canine models of heart failure/cardiomyopathy have demonstrated that GLP-1 administration is associated with improved cardiac output, decreased LV end-diastolic volume and reduced myocyte apoptosis [29]. Furthermore, mice lacking the GLP-1 receptor were reported to have LV diastolic dysfunction, greater LV wall thickening and impaired LV contractile function [30].

However, human studies confirming this link between GLP-1 treatment and cardiac function and structure are limited. In a small study of 12 patients with CHF, LV function as well as their exercise capacity improved after 5 weeks treatment with GLP-1 therapy [27]. Similarly, 72 h of GLP-1 infusion was associated with improved LV ejection fraction in survivors of acute myocardial infarction [26] and GLP-1 analogue (exenatide) administered during percutaneous coronary intervention reduced reperfusion injury and infarct size [31]. Although promising, these studies are limited by their small sample size and the non-randomised methodology. However several randomised trials have recently been published. In one study 20 patients were randomised to GLP-1 or saline infusion after percutaneous coronary intervention; GLP-1 was found to ameliorate LV dysfunction [32]. Another randomised cross-over study demonstrated that GLP-1 therapy improved LV function in 14 patients with coronary artery disease [33]. Whilst the above findings are highly promising, data are lacking around the efficacy of GLP-1 therapy at improving cardiac function in high risk populations without overt cardiovascular disease. This limitation is particularly relevant to young individuals with T2DM who are likely to have extreme levels of obesity and present with sub-clinical diastolic dysfunction. Therefore research is needed to investigate to what extent GLP-1 therapy can ameliorate the early stages of cardiac dysfunction.

## Methods

### Aims

The aims of the study are to evaluate the effect of liraglutide on cardiac structure and function in younger adults with T2DM compared to sitagliptin and assess the effects on glycometabolic control and aerobic fitness. The primary outcome measure is the PEDSR, measured by CMR at baseline and 26 weeks. Other measures of cardiac structure and function assessed in the study and secondary outcome measures are illustrated in Additional file 1: Appendices 1 and 2.

The primary hypothesis is that treatment with liraglutide, GLP-1 analogue, for 26 weeks lead to improvement in PEDSR in younger adults with T2DM compared to the clinically relevant active comparator sitagliptin, a DPP-4 inhibitor.

### Study design

This is a prospective, randomised, open-label, blind end-point (PROBE) active-comparator trial. Individuals will be randomised to receive a maintenance dose of liraglutide 1.8 mg daily or sitagliptin 100 mg daily. Study outcomes will be assessed and analysed by individuals blinded to intervention allocation.

### Study population

Potential participants will be identified from both primary and secondary care in Leicestershire using electronic databases. All patients meeting the inclusion and exclusion criteria (Table 1) will be invited for screening and those who remain eligible after screening will proceed to visit 1–6 as outlined in Additional file 1: Appendix 3.

**Table 1:** Inclusion and exclusion criteria

*Inclusion criteria*
Capacity to provide informed consent before any trial-related activities
Individuals aged 18–50 years inclusive
Established T2DM
BMI ≥30 kg/m^2^ (≥27 kg/m^2^ for South Asians or other BME populations)
On mono or combination oral OAD therapy (sulphonylurea and/or metformin) for ≥ 3 months
No prescribed thiazolidinediones within the last 3 months
An HbA1c value of greater than or equal to 6.5 % and less than 10 % (venous blood)
*Exclusion criteria*
<18 years old
Absolute contraindications to MRI
Type 1 diabetes (identified through C-peptide analysis in cases of diagnostic uncertainty)
Females of child bearing potential who are pregnant, breast-feeding or intend to become pregnant or are not using or willing to use adequate contraceptive methods
Suffer from terminal illness
Have impaired renal function (eGFR <30 ml/min/1.73 m^2^)
Impaired liver function (ALT ≥2.5 times upper limit of normal)
Known Hepatitis B or Hepatitis C
Clinically significant active cardiovascular disease including history of myocardial infarction within the past 6 months and/or heart failure (NYHA class III and IV) at the discretion of the investigator
Recurrent major hypoglycaemia as judged by the investigator
Known or suspected allergy to the trial products
Known or suspected thyroid disease (such as thyroid nodule or thyroid cancer)
Receipt of any investigational drug within 4 months prior to this trial
Have severe and enduring mental health problems
Are not primarily responsible for their own care

### Study visits

The outline of the study visits and measures taken is shown in Additional file 1: Appendix 4.

#### Visit 0—screening visit

The study clinician will check eligibility of the potential participants and obtain written informed consent. Following this, a preliminary ‘on the spot’ capillary test for HbA1c using a near patient testing kit will be carried out. Those participants with on the spot HbA1c of either <6 or >11 % will be informed on the day that they are not eligible to continue. However, those with preliminary on the spot HbA1c ≥6 and ≤11 % will continue through to baseline visit 1, to have their eligibility confirmed with venous HbA1c. Eligible participants will proceed for baseline measures (visit 1).

#### Baseline and follow-up data collection visits

Participants will attend 6 data collection visits in total during the study over a period of approximately 6 months.

#### Visit 1 (baseline visit)

Baseline data including patients’ demographics, medical history and anthropometric measures (weight, height, waist and hip measure, percentage body fat, muscle mass, fat free mass and visceral rating) will be collected. Participants will be asked to complete questionnaires about their wellbeing and satisfaction with their diabetes treatment. Blood samples will also be taken to check a range of baseline measures including diabetes control, lipid profile and measures of inflammation and immune function. All females of child bearing potential will have pregnancy test and assurance of adequate use of contraception will be obtained.

#### Visit 2 (baseline MRI visit)

Participants will have a comprehensive stress CMR scan performed at visit 2 as outlined below. Information about the MRI test will have been given to the participant before they join the study. Patients who are unable to complete the CMR will remain in the study for secondary end points.

#### Visit 3 (week 0)—randomisation visit

Prior to randomisation, participants will undergo a maximum incremental exercise test on a stationary bicycle (electromagnetically braked cycle ergometer) with expired gas analysis to determine maximal oxygen consumption (VO_2max_), the gold standard assessment of cardiovascular fitness. They will then be randomly assigned to either liraglutide or sitagliptin (1:1) using an independent online computerised randomisation system. Up-titration of liraglutide dose will occur at week 1(0.6 mg), week 2 (1.2 mg) and week 3 (maintenance dose of 1.8 mg). Monitoring and adjusting background medication are discussed in the following section under study medication. Follow up telephone contact will be made to check participant’s drug tolerance and report any adverse events between visit 3 and 4.

#### Visit 4 (week 12)

First follow up will be conducted at 12 weeks. Measures taken during visit 1 will be repeated.

#### Visit 5–6 (week 26)

The CMR scan and measures taken at visit 1 and 3 will be repeated at the final two follow-up visits. Visit 6 will be the end of study.

### Study medications

Name: Liraglutide (Victoza) or Sitagliptin (Januvia).

Pharmaceutical form: Solution for liraglutide and tablet for sitagliptin.

Pharmaceutical dosage and accountability: liraglutide will be self-administered as subcutaneous injection initiated at 0.6 mg daily in week 1, titrated up to 1.2 mg daily in week 2 and maintenance dose of 1.8 mg thereafter until completion of the study as advised per the study team. Sitagliptin dose will be self-administered orally 100 mg daily. Dose of both medications will be lowered to the maximum tolerated dose at the investigator discretion. Local NHS Trust pharmacies will be supplying the sitagliptin whilst Novo Nordisk will be supplying the liraglutide, which will be dispensed by the UHL pharmacy.

#### Background medication and glycaemic control

Use of metformin or sulphonlyurea (SU) will be considered as background medication (non-investigational medicinal product). Participants treated with either metformin or SU at inclusion into the study will continue their pre-study dose throughout the study. If the HbA1c level falls <7 % (58 mmol/l), SU dose will be halved at baseline or stopped during the study if hypoglycaemia develops. Other dose reductions in SU therapy due to hypoglycaemia will be allowed at the discretion of the principle investigator.

#### Rescue medication

The criterion for starting glycaemic rescue therapy is a fasting plasma glucose value of 11.1 mmol/l at 12 weeks. Rescue therapy initiated will be determined by the investigator as clinically appropriate, either up titration of current anti-hyperglycaemic agents (AHA) or the stepwise addition of non-insulin AHA(s) and then insulin therapies. All patients receiving rescue therapy will be followed up until the end of the study.

### MRI

**Fig. 1:**
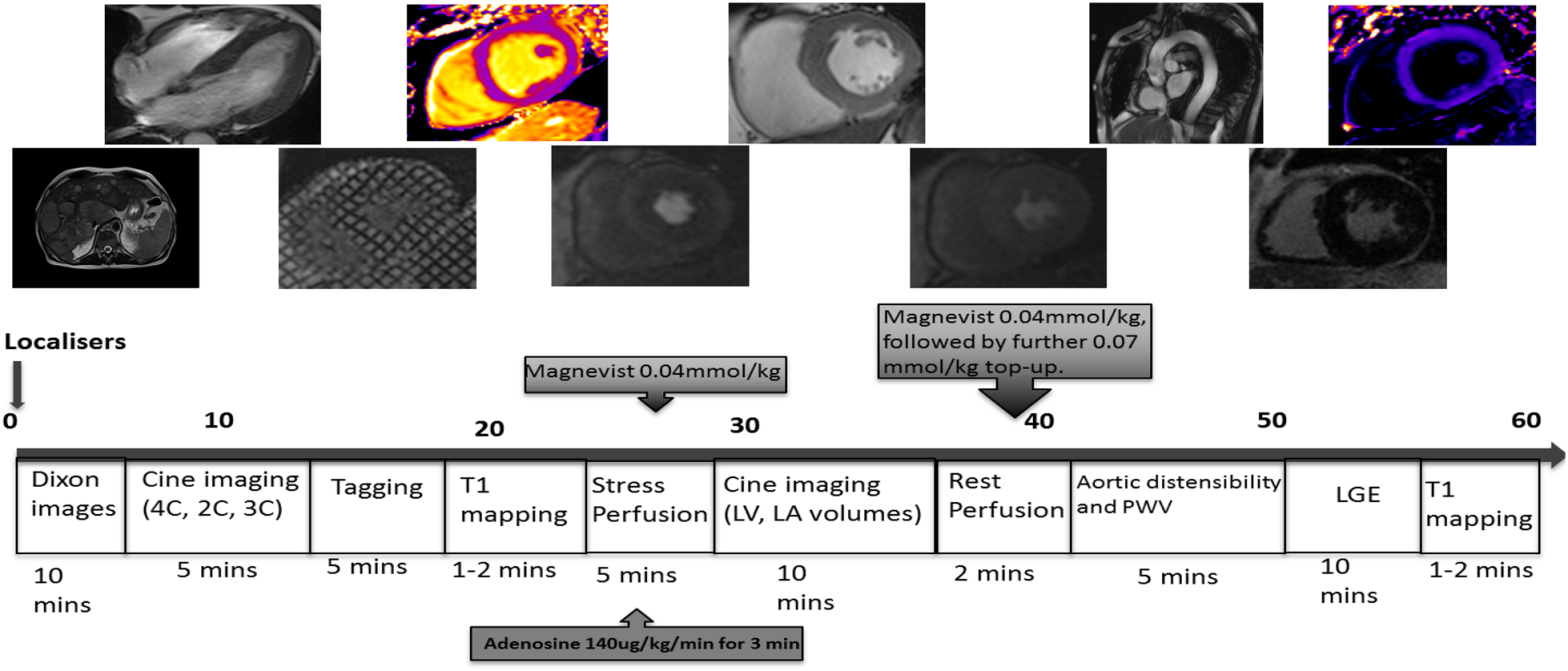
Cardiac magnetic resonance imaging (CMR) protocol. CMR protocol used (*LA* left atrium, *LV* left ventricle, *LGE* late gadolinium enhancement)

The MRI protocol is outlined in Fig. 1 and is similar to that previously described [34] but at 1.5 T (Aera, Siemens, Erlangen), as the reproducibility of PEDSR appears to be better at this field strength than at 3T [35]. In addition thoracic and abdominal subcutaneous and visceral fat assessment using Dixon gradient-echo pulse sequence will be undertaken [36]. After localisers, Dixon sequences will be acquired covering the thorax and abdomen. Steady-state free precession cine images will be acquired in 4-, 3-, and 2-chamber views. Myocardial tissue tagging will be performed in three short axis slices to assess pre and post contrast (15 min) T1 maps will be obtained in a mid-short axis slice to determine gadolinium volume of distribution correcting for haematocrit [37, 38]. Perfusion images will be acquired after pharmacological vasodilation with adenosine, 140 μg/kg/min, for 2–3 min and during data acquisition until a haemodynamic response is achieved (a drop in blood pressure of 10 mmHg and/or a rise in heart rate of 10 bpm). A gadolinium-based contrast agent (Magnevist, Bayer Healthcare, Leverkusen, Germany) will be administered intravenously (0.04 mmol/kg) at 5 ml/s, followed by a 20 ml saline flush. First-pass perfusion will be assessed for three slices (basal, mid, and apical) acquiring every heartbeat using a saturation recovery gradient echo sequence. Rest imaging will be performed approximately 10 min after stress imaging with a further 0.04 mmol/kg of contrast. In the intervening time, a stack of short-axis slices will be obtained using cine imaging covering the entire left LV. A further 0.07 mmol/kg of contrast will be given to bring the total dose to 0.15 mmol/kg. At least 10 min after this, late gadolinium enhancement images will be obtained with the use of an inversion-recovery prepared, segmented gradient echo sequence, as previously described.

#### CMR analysis

MRI scans will be anonymised and sent to a standalone work station for blinded analysis as previously described [18, 35, 39]. Briefly strain and PEDSR will be quantified from tagging and cine images using InTag and feature tracking respectively. LV volumes, mass and function will be calculated using commercially available non-propriety software. Interobserver and intraobserver variability will be calculated on at least 10 random datasets by two experienced observers and results reported. The epicardium and endocardium will be contoured on the perfusion images, along with a region of interest in the LV blood pool, to generate signal intensity curves. MPR will be calculated using Fermi-constrained deconvolution as previously described [18]. Pre and post contrast (15 min) T1 maps will be obtained in a mid-short axis slice to determine gadolinium volume of distribution correcting for haematocrit [40]. Late gadolinium enhancement will be assessed qualitatively as absent or present and quantified by a thresholding technique >5 standard deviations above remote normal myocardium [39].

### Statistics

#### Sample size

The primary aim is to detect a difference in peak diastolic strain rate 0.2/s with a standard deviation of 0.3/s from baseline to completion of the study (26 weeks). This was based on recently published study of obese young subjects with T2DM [13]. With power of 80 % and a 2 sided significance of 0.05, the sample size analysis requires 36 participants per group to complete the study. Allowing for a 20 % drop-out, we will aim to recruit 45 participants per group.

#### Statistical methods and analysis

Baseline characteristics will be displayed by group as mean and standard deviation (or median interquartile range for non-normalised data) for continuous variables and counts and percentages for categorical. The distribution of all primary and secondary variables will be assessed. Where data are found to be non-normally distributed, transformations will be applied. The primary outcome will be compared by treatment group using analysis of covariance (ANCOVA) modelling, adjusted for baseline value; the mean difference between the two groups and the 95 % confidence interval will be presented. A similar analysis will be used for all continuous secondary outcomes. Categorical outcomes will be compared by group using logistical regression analysis. All statistical tests will be 2 sided and p < 0.05 will be considered statistically significant.

Participants randomised in the study will be analysed according to intention to treat (ITT) principle. Per-protocol analysis will be carried out as a sensitivity analysis for only those who are compliant to the treatment. We will also attempt to address bias by comparing the characteristics of those with missing outcome data to those who have completed follow up.

### Ethical issues

The study is conducted in accordance with the principles of the 1996 Helsinki Declarations, ICH-GCP guidelines. Ethical approval as a CTIMP was granted by NRES committee West midlands (13/WM/0311).

### Trial oversight and governance

The study sponsor is the University of Leicester and is managed by the Leicester clinical trial unit (CTU) and is overseen by trial steering committee (TSC) comprised of independent chair and a group of experts. This study is registered on http://www.clinicaltrials.gov (NCT02043054) and monitored by the University of Leicester.

Safety of the participants will be independently monitored by the Data monitoring and Ethics Committee (DMEC), which will make recommendations to the TSC for appropriate action in the interest of the participants.

### Current status and time scale

Recruitment started in January 2014 and the last patient follow up should complete by June 2016. To date, 103 patients have been screened, of which 77 were eligible to participate and 70 have been randomised to the study.

## Discussion

Heart failure is one of the main determinants of long term mortality in patients with T2DM, and subtle abnormalities in diastolic function have been reported even at early stage of the disease [13]. Once CHF is established it is associated with a poor prognosis [41]. There is therefore a pressing need to identify therapies that may modify the disease process in T2DM and prevent longer term cardiac complications.

The benefits of lifestyle interventions such as weight loss and exercise on glycaemic control have been reported in several large prospective trials. Indeed, complete reversal of T2DM has been shown following bariatric surgery [42, 43]. However there is limited data currently on the effects on cardiovascular outcomes. The use of a 471 kcal/day low energy diet in patients with T2DM has been shown to be associated with improved diastolic filling on CMR [44], and another study suggested a benefit in diastolic parameters following 3 months of aerobic exercise [45]. Both of these studies were small, single centre pilots.

Currently, the evidence for an effect of any glucose lowering therapy on cardiovascular outcomes is inconsistent, and until recently only metformin (from the UKPDS trial) had been shown to have any benefit [46]. The recent expansion in classes of hypoglycaemic agents has led to several clinical trials specifically evaluating the efficacy of these drugs for beneficial effects on cardiovascular endpoints, which have generally demonstrated neutral effects. The trial evaluating cardiovascular outcomes with sitagliptin (TECOS), saxagliptin assessment of vascular outcomes recorded in patients with diabetes mellitus (SAVOR-TIMI 53) and examination of cardiovascular outcomes: alogliptin (EXAMINE) studies all demonstrated neutral effect on major cardiovascular events with DPP-4 inhibitors, sitagliptin, saxagliptin and alogliptin [47–49]. Similarly, the ELIXA (evaluation of cardiovascular outcomes in patients with type 2 diabetes after acute coronary syndrome during treatment with lixisenatide) trial, which examined the GLP-1 agonist lixisenatide versus placebo in patients with T2DM and a history of recent acute coronary syndrome, found a neutral effect of the drug on adverse cardiovascular events [50]. Most recently, the LEADER (liraglutide effect and action in diabetes: evaluation of cardiovascular outcome results—a long term evaluation) trial demonstrated the cardiovascular benefit of liraglutide in high-risk cardiovascular patients with T2DM [51]. In fact, liraglutide has demonstrated superiority to placebo with a 13 % relative risk reduction in the first occurrence of cardiovascular death, nonfatal MI or nonfatal stroke and 22 % reduction in mortality due to cardiovascular causes. This is the very first cardiovascular safety trial among incretin-based therapies that has demonstrated positive outcome on cardiovascular effect. A another group of new therapeutic agents, SGLT-2 inhibitors has also demonstrated a 38 % relative risk reduction in mortality due to cardiovascular causes in the empagliflozin cardiovascular outcome event trial in type 2 diabetes mellitus patients trial (EMPA-REG) [52].

However, there were concerns about the unexpected increased risk of hospitalisation from heart failure in the cohort treated with DPP-4 inhibitor, saxagliptin in the SAVOR-TIMI 53 study. The vildagliptin in ventricular dysfunction diabetes (VIVIDD) trial demonstrated statistically significant increase in LV endiastolic volume and a trend towards an increase in LV systolic volume associated with vildagliptin [53]. The other studies TESCOS and ELIXA trials showed neutral effect on hospitalisations for heart failure [47, 50]. In the recently published LEADER study, a lower risk of hospitalisation from heart failure was shown in the liraglutide group although statistically not significant [51]. In general, retrospective data has suggested a possible beneficial effect of GLP-1 agonists on cardiac function in T2DM [54]. The exact mechanism of how the incretin based therapies affect cardiac function was not clearly understood. Although not necessarily a class effect, the investigators believed that the LYDIA study was well placed at the time to examine changes in cardiac parameters and function following treatment with a DPP-4 inhibitor and that sitagliptin as the most widely used DPP-4 inhibitor would be a suitable comparator. To the best of our knowledge, this will be the first study in the UK to establish the impact of liraglutide on cardiac structure and function using CMR in younger adults with T2DM. The study aims to comprehensively describe changes in various parameters of cardiac structure and function in patients treated with liraglutide compared to sitagliptin.

There are limitations in this study. As the study is of relatively short duration, no long-term data will be available from the study beyond 6 months. Secondly, the lack of placebo may inadvertently over accentuate the improvement seen with liraglutide, if the DPP-4 inhibitors used as a comparator indeed depress myocardial performance. However, the results will inform us if treatment with liraglutide can actually improve diastolic function and if so, to what extent in younger adults with T2DM. This will be of clinical relevance to decide management strategies to ameliorate the progress of early stages of cardiac dysfunction in the future, and if positive would provide justification for longer term, prospective trials on the potential benefits of GLP-1 agonists to prevent heart failure in this population.

## Additional file

10.1186/s12933-016-0421-6 Appendices.

## Abbreviations

ANCOVA: analysis of covariance
AHA: antihyperglycaemic agent
CABG: coronary artery bypass graft
CHF: chronic heart failure
CMR: cardiac magnetic resonance imaging
CTU: clinical trial unit
CVD: cardiovascular disease
DPP-4 enzyme: depeptidyl peptidase-4 enzyme
DMEC: Data Monitoring and Ethics Committee
EF: ejection fraction
HF-NEF: heart failure with normal ejection fraction
ELIXA: evaluation of cardiovascular outcomes in patients with type 2 diabetes after acute coronary syndrome during treatment with lixisenatide
EMPA-REG: empagliflozin cardiovascular outcome event trial in type 2 diabetes mellitus patients
GLP-1: glucagon like peptide-1
GLP-1 R: glucagon like peptide-1 receptor
HbA1c: glycated haemoglobin A1c
hs CRP: highly sensitive CRP
ITT: intention to treat
LV: left ventricle
MRI: magnetic resonance imaging
NDA: National Diabetes Audit
NRES: National research ethics service
PEDSR: peak early diastolic strain rate
SGLT-2: sodium glucose co-transporter-2
SU: sulphonylurea
RCT: randomised controlled trial
T2DM: type 2 diabetes
TSC: trial steering committee
UHL: University Hospitals of Leicester
VO_2max_: maximal oxygen consumption
