# Supplementary material for: Rationale and design of the randomised controlled trial to assess the impact of liraglutide on cardiac function and structure in young adults with type 2 diabetes (the LYDIA study)
Source: Cardiovasc Diabetol. 2016 Jul 21;15:102. doi: 10.1186/s12933-016-0421-6 (PMC4955217; doi:10.1186/s12933-016-0421-6)
Supplement: Supplementary file 1 — 10.1186/s12933-016-0421-6 Appendices. [file 12933_2016_421_MOESM1_ESM.docx]

**Appendix 1**. Other cardiac measures of function and structure

| - Peak Systolic Strain - Left Ventricular Ejection Fraction - Stroke volume - LV end-diastolic volume - LV end-systolic volume - LV end-diastolic mass - Left Ventricular End Diastolic Mass/volume ratio - Pre-and post contrast T1 mapping to calculate volume of distribution, a marker of diffuse cardiac fibrosis - Myocardial Perfusion Reserve ( a measure of microvascular function) |
| --- |

**Appendix 2 –** Secondary outcome measures

| - HbA1c - Lipid profile including total-, LDL- and HDL-cholesterol and triglycerides - Liver Function Tests - Renal Function Tests - Thyroid function tests - Complete Blood Count (Hematocrit) - Vitamin D - Endothelial function (EPCs & SDF-1α) - Measures of inflammation (hs-CRP) - Anthropometirc measures – body weight, height, BMI, blood pressure - Cardiorespiratory fitness (V02 max) - Level of physical activity (accelerometer/inclinometer) - 7-point glucose profile - Treatment satisfaction & quality of life - Thoracic and abdominal subcutaneous and visceral fat assessment |
| --- |

**Appendix 3** – Participant flow chart

**
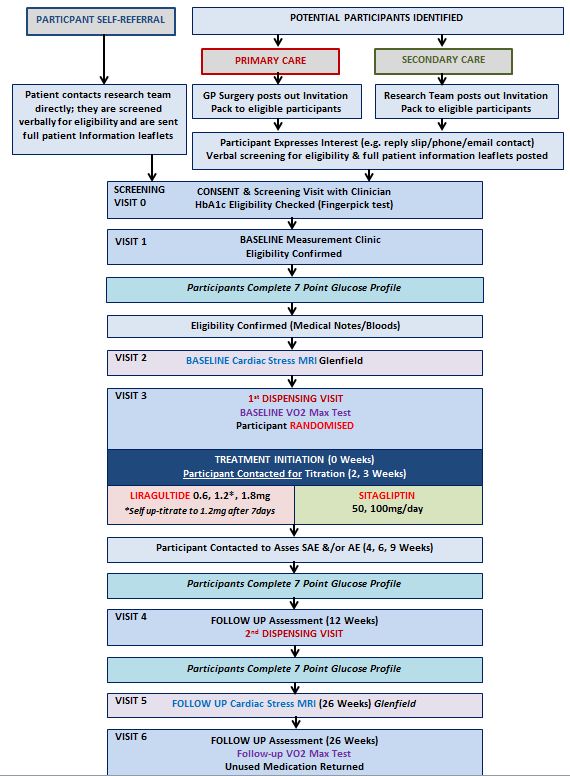
**

**Appendix 4** – Examination and measures

| Visit | Consent /Screening (Visit 0) | Baseline (Visit 1) | Baseline (Visit 2) | Baseline (Visit 3) | Follow Up Contact | Follow Up Contact | Pre-Visit 4 Self Reporting | 1^st^ Follow Up, 2^nd^ Dispensing (Visit 4 ) | Pre-Visit 6 Self Reporting | Follow Up MRI (Visit 5) | 2^nd^ Follow Up (Visit 6) |
| --- | --- | --- | --- | --- | --- | --- | --- | --- | --- | --- | --- |
| Time-point | n/a | n/a | n/a | Week 0 | Weeks 1,2,3 | Weeks 4,6,9 | 11 weeks* | 12 weeks | 25 weeks* | 26 weeks  (< or > v6) | 26 weeks  (< or > v5) |
| Visit Window | n/a | n/a | n/a | n/a | +/- 7 days | +/- 7 days | +/- 7 days | +/- 14 days | +/- 7 days | +/- 14 days | +/- 14 days |
| HbA1C Finger prick Test | √ |  |  |  |  |  |  |  |  |  |  |
| Cardiac MRI |  |  | √ |  |  |  |  |  |  | √ |  |
| Standard Biochemical variables |  | √ |  |  |  |  |  | √ |  |  | √ |
| Other Biochemical measures i.e. chronic low-grade inflammation |  | √ |  |  |  |  |  | √ |  |  | √ |
| Anthropometric variables |  | √ |  |  |  |  |  | √ |  |  | √ |
| Blood Pressure |  | √ |  |  |  |  |  | √ |  |  | √ |
| Smoking, Medical & Family History |  | √ |  |  |  |  |  |  |  |  |  |
| Pregnancy Test |  | √ |  | √ |  |  |  | √ |  |  | √ |
| Endothelial function |  | √ |  |  |  |  |  |  |  |  | √ |
| Vitamin D |  | √ |  |  |  |  |  |  |  |  | √ |
| Cardio-respiratory fitness (graded VO2 max test) |  |  |  | √ (+2 weeks) |  |  |  |  |  |  | √ |

| Visit | Consent /Screening (Visit 0) | Baseline (Visit 1) | Baseline (Visit 2) | Baseline (Visit 3) | (Follow Up Contact | (Follow Up Contact | Pre-Visit 4 Self Reporting | 1^st^ Follow Up, 2^nd^ Dispensing (Visit 4 ) | Pre-Visit 6 Self Reporting | Follow Up MRI (Visit 5) | 2^nd^ Follow Up (Visit 6) |
| --- | --- | --- | --- | --- | --- | --- | --- | --- | --- | --- | --- |
| Time-point | Any | n/a | n/a | Week 0 | Weeks 1,2,3 | Weeks 4,6,9 | 11 weeks* | 12 weeks | 25 weeks* | 26 weeks  (< or > v6) | 26 weeks (< or > v5) |
| Visit Window | n/a | n/a | n/a | n/a | +/- 7 days | +/- 7 days | +/- 7 days | +/- 14 days | +/- 7 days | +/- 14days | +/- 14 days |
| Physical Activity and Sitting Time** |  | √ |  |  |  |  |  | √ |  |  | √ |
| Quality of life and depression |  | √ |  |  |  |  |  | √ |  |  | √ |
| Treatment satisfaction |  | √ |  |  |  |  |  | √ |  |  | √ |
| 7-point G profile* |  |  | √ |  |  |  | √ |  | √ |  |  |
| Hypoglycaemia Diary*** |  |  |  |  |  |  |  | √ |  |  | √ |
| Adverse events |  | √ |  |  | √ | √ |  | √ |  |  | √ |

**Key:** (*) The 7-point glucose profile is self-reported by the participant at (-)2 weeks, 11 weeks and 25 weeks prior to the visits 3, 4 and 6 in which the data is collected.

(**) An accelerometer and inclinometer are setup for 7 days at visit 1 to be returned in the post or at visit 3 and then setup at visits 4 and 6 and returned in the post.

(***) The hypoglycaemia diary is self-reported by the patient throughout the 26 weeks of drug treatment and a copy of this taken at visit 4 and visit 6.
